# Caesarean section rates from Malaysian tertiary hospitals using Robson’s 10-group classification

**DOI:** 10.1186/s12884-020-2760-2

**Published:** 2020-01-31

**Authors:** Shamala Devi Karalasingam, Ravichandran Jeganathan, Ravindran Jegasothy, Daniel D. Reidpath

**Affiliations:** 1 National Obstetrics Registry, Institute Clinical Research, National Institute of Health, No 1 Jalan Setia Murni U13/52, Seksyen U13, Setai Alam, Shah Alam, 40170 Selangor Malaysia; 2 Sultanah Aminah Hospital, J1, Jalan Abu Bakar, 80000 Johor Bahru, Johor Malaysia; 3 Faculty of Medicine, MAHSA University, Jalan SP2, Bandar Saujana Putra, 42610 Jenjarom, Selangor Malaysia; 4 South East Asia Community Observatory (SEACO), 125, Jalan Sia Her Yam, Kampung Abdullah, 85000 Segamat, Johor Malaysia; 5 Jeffrey Cheah School of Medicine and Health Sciences, Monash University Malaysia, 47500 Subang Jaya, Malaysia

**Keywords:** Robson’s classification, Caesarean section, Birth registry

## Abstract

**Background:**

Rising caesarean section rates is a concern worldwide. This study aimed to use Robson’s ten group classification to identify which groups of women were contributing most to the rising caesarean section rates in Malaysian tertiary hospitals and to compare between hospitals, using a common standard set of variables.

**Methods:**

A 5-year (2011–2015) cross-sectional study was conducted using data from the Malaysian National Obstetrics Registry (NOR). A total of 608,747 deliveries were recorded from 11 tertiary state hospitals and 1 tertiary hospital from the Federal territory.

**Results:**

During the study period, there were 141,257 Caesarean sections (23.2%). Caesarean sections in Group 1 (nulliparous term pregnancy in spontaneous labour) and Group 3 (multiparous term pregnancy in spontaneous labour) had an increasing trend from 2011 to 2015. The group that contributed most to the overall caesarean section rates was Group 5 (multiparous, singleton, cephalic≥37 weeks with previous caesarean section) and the rates remained high during the 5-year study period. Groups 6, 7 and 9 had the highest caesarean section rates but they made the smallest contribution to the overall rates.

**Conclusions:**

Like many countries, the rate of caesarean section has risen over time, and the rise is driven by caesarean section in low-risk groups. There was an important hospital to hospital variation. The rise in caesarean section rates reflects a globally disturbing trend, and changes in policy and training that creates a uniform standard across hospitals should be considered.

## Synopsis

The rise in Caesarean section (CS) reflects a globally disturbing trend. Changes in policy and training that creates a uniform standard across hospitals should be considered.

## Background

In 1985 the World Health Organization (WHO) set the optimal rate for Caesarean section (CS) at 10–15% of all births [1]; and notwithstanding this ideal, for the last quarter of a century, CS rates have been increasing. A recent review showed a global CS rate around 18.6% with some regional rates above 27.2% [2]. For example, in recent years the CS rates in Denmark Ireland, and Turkey, were 20.6, 26, and 42.7% respectively [3]. In Lithuania the CS rates have increased more than 2.5 fold from 9.6% in 1995 to 25% in 2011 [4].The rise in CS rates above the WHO recommendation had been a cause for concern because CS carries inherent risks of mortality and morbidity for both the mother and the baby. If there is an ideal rate, any excess may be indicative of unnecessary medical intervention [2]. In a moderating statement released in 2015, however, WHO stepped back from a fixed, ideal rate and suggested that “every effort should be made to provide caesarean sections to women in need, rather than striving to achieve a specific rate [5].

Determining whether any particular CS is clinically required, is challenging because the decision to perform the procedure often rests on an individual clinical judgement made under significant time constraints. Given that the rate of clinically required CS may vary for demographic reasons between populations. However, one would, on average, expect a similar rate of CS within similar subsets of the same population, with the reasons for CS varying over time [6]. An analysis of Malaysian tertiary hospital data, however, showed substantial variation in CS rates (not accounted for by demographic variation) from as low as 16% to more than 32% [7, 8]. This kind of variation within the context of a single hospital system within one country is a cause for potential concern and invites investigation [9].

Attempts to classify CS has led to 27 separate classificatory systems based on various factors. Factors included (i) clinical indications “such as dystocia, acute intrapartum fetal distress”, (ii) a clinical judgment about the degree of urgency, (iii) features about the mother such as parity or a previous history of CS, and (iv) other approaches including an evaluation of staffing. A recent systematic review of CS classificatory systems concluded that the Robson’s classification provided the best method for collecting useful comparative data [5]. Robson’s classification accounts for fetal presentation, the number of previous pregnancies, the course of the delivery, and gestational age [10]. The system is simple to implement, provides comparable data between settings and over time, and allows for an analysis of the indicators of CS. The approach has been used widely since its publication in 2001 [10]. Recently it has been applied in small pilot settings involving a single small hospital through to larger national studies involving multiple hospitals [11]. It has never been applied to Malaysian data and rarely involving the number of births recorded in the Malaysian National Obstetrics Registry [7, 8]. Given the disparity in observed hospital CS rates, and the potential to look at changes over time, such an analysis would be timely; and could identify settings where further effort is required to address the CS rate.

## Methods

The Malaysian NOR is a register of births in government tertiary hospitals established in July 2009. It has become one of the world’s largest active birth registries recording maternal details, previous obstetric history, and birth outcomes. The NOR records all births (live births and stillbirths) at ≥22 weeks gestation. A complete description of the NOR can be found in the annual reports [7, 8] and the website [http://www.acrm.org.my/nor/]. Ethical approval for the NOR was provided by the Medical Research and Ethics Committee of the Ministry of Health, Malaysia (Approval number: NMRR15–620-25,530).

**Fig. 1:**
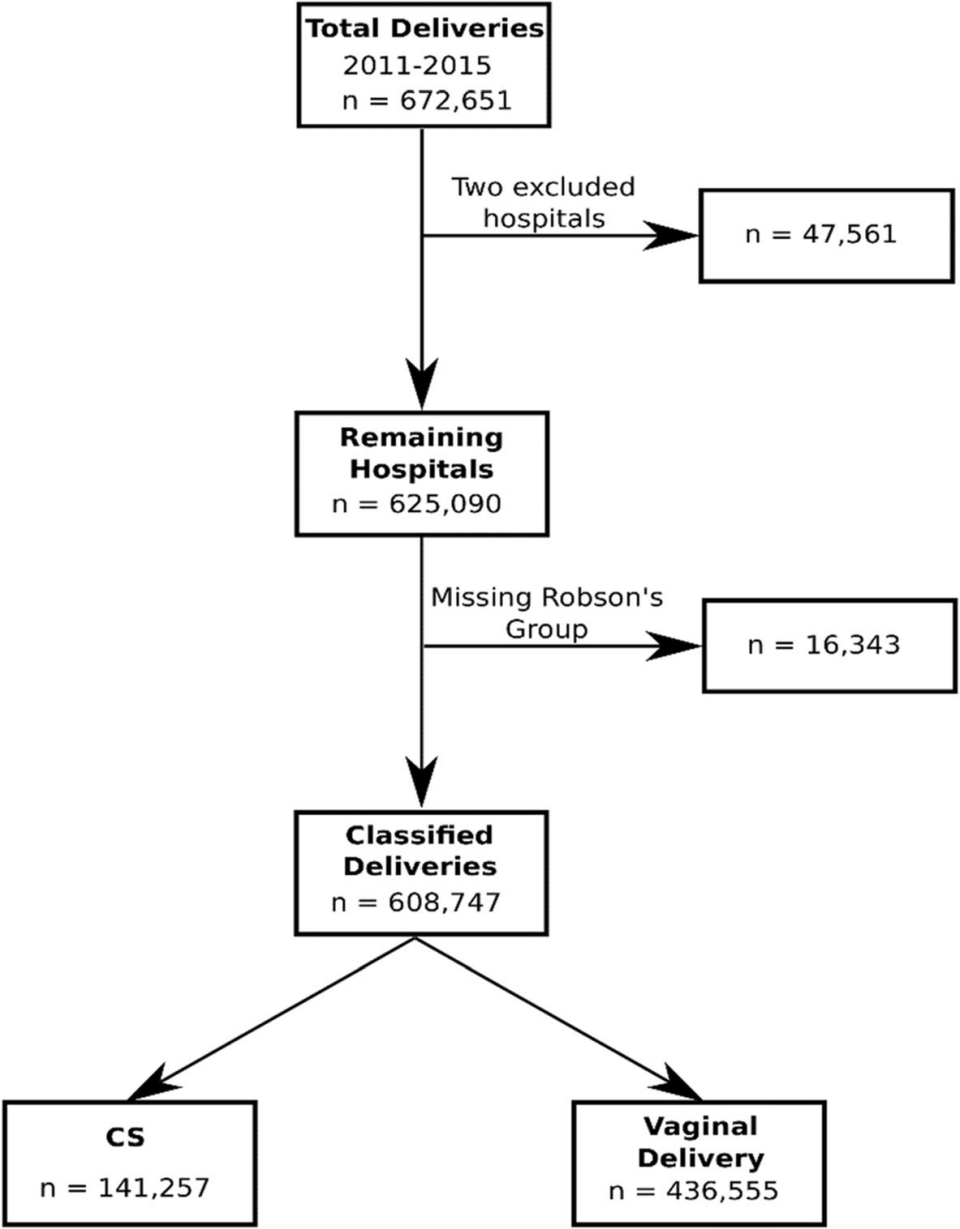
The flow chart in this study

Data for this study were contributed between 1 January 2011 and 31 December 2015 by 11 of the 13 tertiary state government hospitals in the NOR, as well as the main tertiary hospital in the Federal Territory of Kuala Lumpur (i.e., 12 hospitals in all). Two of the tertiary state government hospitals were excluded from this analysis because there were some concerns about the completeness of elements of the data. Fortunately, these two hospitals make only a modest contribution to the total NOR data; see Fig. 1. In the final analytic dataset, there was a total of 608,747 deliveries, of which there were 141,257 CS – an annual average rate of 23.2%. The births were classified according to Robson groups, which allocates each birth to one of 10 discreet, non-overlapping classes. The descriptors for each Robson group are shown in Table 1 [10]. We sometimes write about “total deliveries” and “total CS”. This refers to all classified deliveries and CS. That is, the reference is to all deliveries/CS for which a Robson group could be assigned. We do not count the missing data in our use of “all”, and the implications of this are discussed later.

**Table 1:** Descriptors for the 10 Robson’s groups

Robson’s Group	Description
1	Nulliparous, singleton, cephalic, ≥ 37 weeks, spontaneous labour
2	Nulliparous, singleton, cephalic, ≥ 37 weeks, induced labour or CS before labour
3	Multiparous women, singleton, cephalic, ≥ 37 weeks, without a previous CS, spontaneous labour
4	Multiparous, singleton, cephalic, ≥ 37 weeks, without a previous uterine scar, induced labour or by CS before labour
5	Multiparous, singleton, cephalic, ≥ 37 weeks, with a previous CS
6	Nulliparous, singleton, breech
7	Multiparous, singleton, breech
8	Multiple pregnancy including women with a previous CS
9	Singleton, transverse or oblique lie, including women with a a previous CS
10	Singleton, cephalic, < 37 weeks, including women with a previous CS

Analyses were conducted using Stata and Microsoft Excel, and figures were generated using the R Statistical Environment [12]. The analysis of Robson’s classification data relies on simple arithmetic counts and proportions described in the World Health Organization’s, “Robson Classification: Implementation Manual” [13].

## Results

**Fig. 2:**
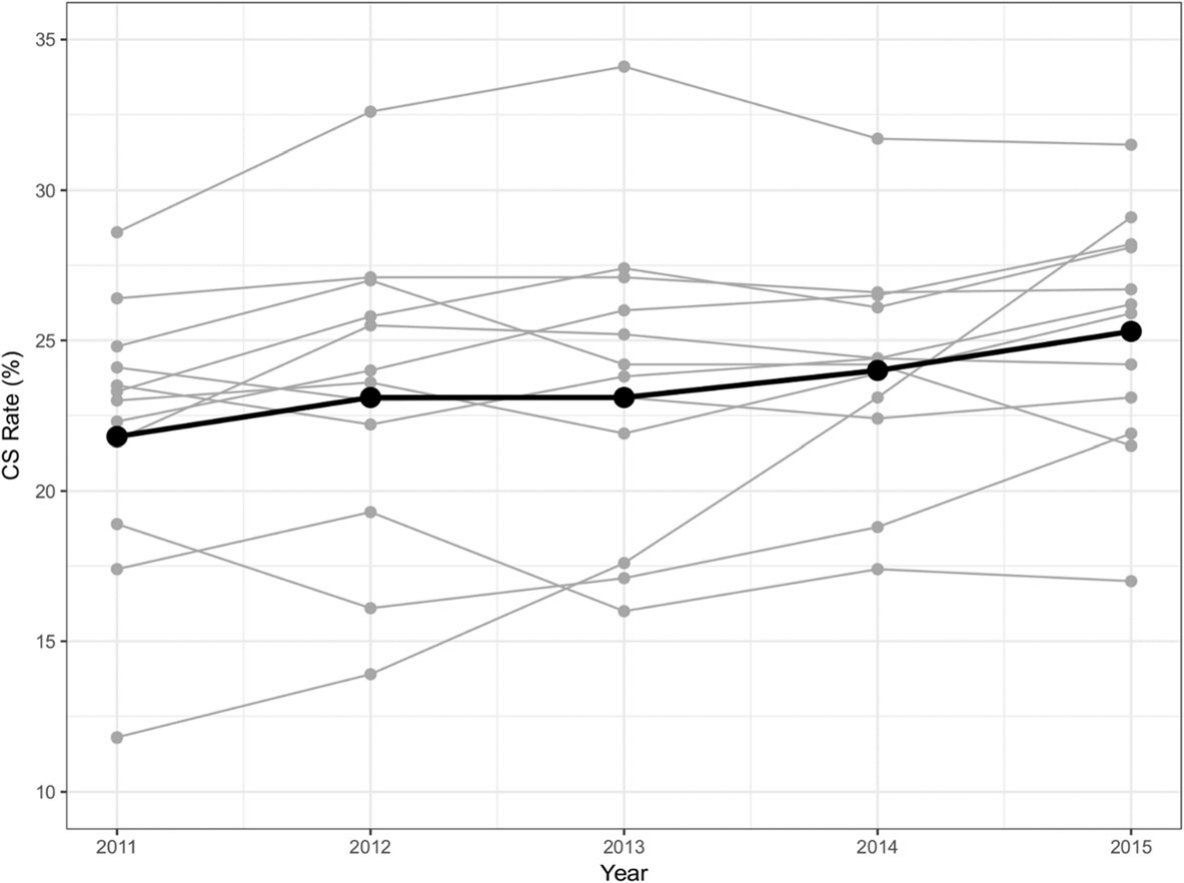
Trend of Caesarean section by participating hospitals from 2011 to 2015

The CS rate in each year for each of the 12 hospitals is shown as grey lines in Fig. 2, with the aggregate hospital rate shown as a black line. There is a clear hospital to hospital variation with some hospitals showing declining CS rates and some with increasing rates. Overall the absolute CS rate has increased 3% over the 5 years from 21.8 to 25.3% (chi-square = 464.7, df = 1, *p* < .0001). The hospital with the highest CS rate lies well above the other hospitals in all years.

Table 2 summarises the aggregated hospital data from all 12 hospitals over the 5 years by Robson group. The columns labelled A to E show: (A) the number of CS in each Robson group; (B) the total number of deliveries in each group; (C) the CS rate within each Robson group (i.e., A/B) as a percentage; (D) the percentage of the total classified deliveries represented by each Robson group (i.e., deliveries for which a Robson’s group was assigned); and (E) the percentage of the total deliveries in each Robson group that were CS. The contribution to the total CS rate by each Robson group is, thus, a function of both the total number of deliveries in each Robson group and the CS rate in that group [14]. The highest rates of CS are found in Robson groups 9 (Singleton, transverse or oblique lie), 6 (Nulliparous, singleton, breech) and 7 (Multiparous, singleton, breech). In group 9, 100% of deliveries were by CS, in group 6, 91.9% of deliveries were by CS, and in group 7, 87.7% of deliveries were by CS. Notwithstanding the very high CS rates in these latter groups, combined they represent only 3.3% of all deliveries and 14% of all CS.

**Table 2:** Rate of Caesarean section by Robson classification group for eleven State and one Federal Territory hospitals in Malaysia, 2011–2015

Robson’s Group	N CS in group	Total N in group	Group Size (%)	Group CS rate (%)	Absolute group contribution to overall CS rate (%)	Relative group contribution to overall CS rate (%)
1	22,111	18,912	19.0	18.6	3.5	15.1
2	15,767	55,600	8.9	28.4	2.5	10.7
3	20,243	214,888	34.4	9.4	3.2	13.8
4	11,157	74,729	12.0	14.9	1.8	7.6
5	28,048	51,096	8.2	54.9	4.5	19.1
6	6120	6657	1.1	91.9	1.0	4.2
7	9091	10,361	1.7	87.7	1.5	6.2
8	6529	12,527	2.0	52.1	1.0	4.4
9	5312	5312	0.8	100.0	0.8	3.6
10	16,879	58,665	9.4	28.8	2.7	11.5
Unclassified	5609	16,343	2.6	34.3	0.9	3.8
Total	146,866	625,090	100.0	23.5	23.5	100.0

1. % = n of women in the group / total N women delivered in the setting × 100

2. % = n of CS in the group / total N of women in the group × 100

3. % = n of CS in the group / total N of women delivered in the setting × 100

4. % = n of CS in the group / total N of CS in the setting × 100

Other groups with higher absolute numbers of CS have lower CS rates. Group 5 (Multiparous, singleton, cephalic, ≥ 37 weeks, with a previous CS) has a high CS rate (54.9%) and the greatest absolute numbers of CS (*n* = 28,048). Group 1 (Nulliparous, singleton, cephalic, ≥ 37 weeks, spontaneous labour) was the second most frequent Robson group (*n* = 22,111) and had a CS rate of 18.6%. The third most frequent, and lowest risk, Robson group (Group 3, Multiparous women, singleton, cephalic, ≥ 37 weeks, without a previous CS, spontaneous labour), has the lowest rate of CS, 9.4%. This result is very likely to reflect an undercount of the rate of repeat CS,] and this is discussed further in the limitations [13].

Ranked in order of their contribution to the overall CS rate (and excluding the unclassified deliveries), the largest contribution is made by Group 5 – a function of a high CS rate (54.9%) and a substantial number of deliveries (*n* = 51,096). The smallest contribution (i.e., ranked 10th) is the Robson group with a 100% CS rate and a low number of deliveries – group 9 (Singleton, transverse or oblique lie).

**Fig. 3:**
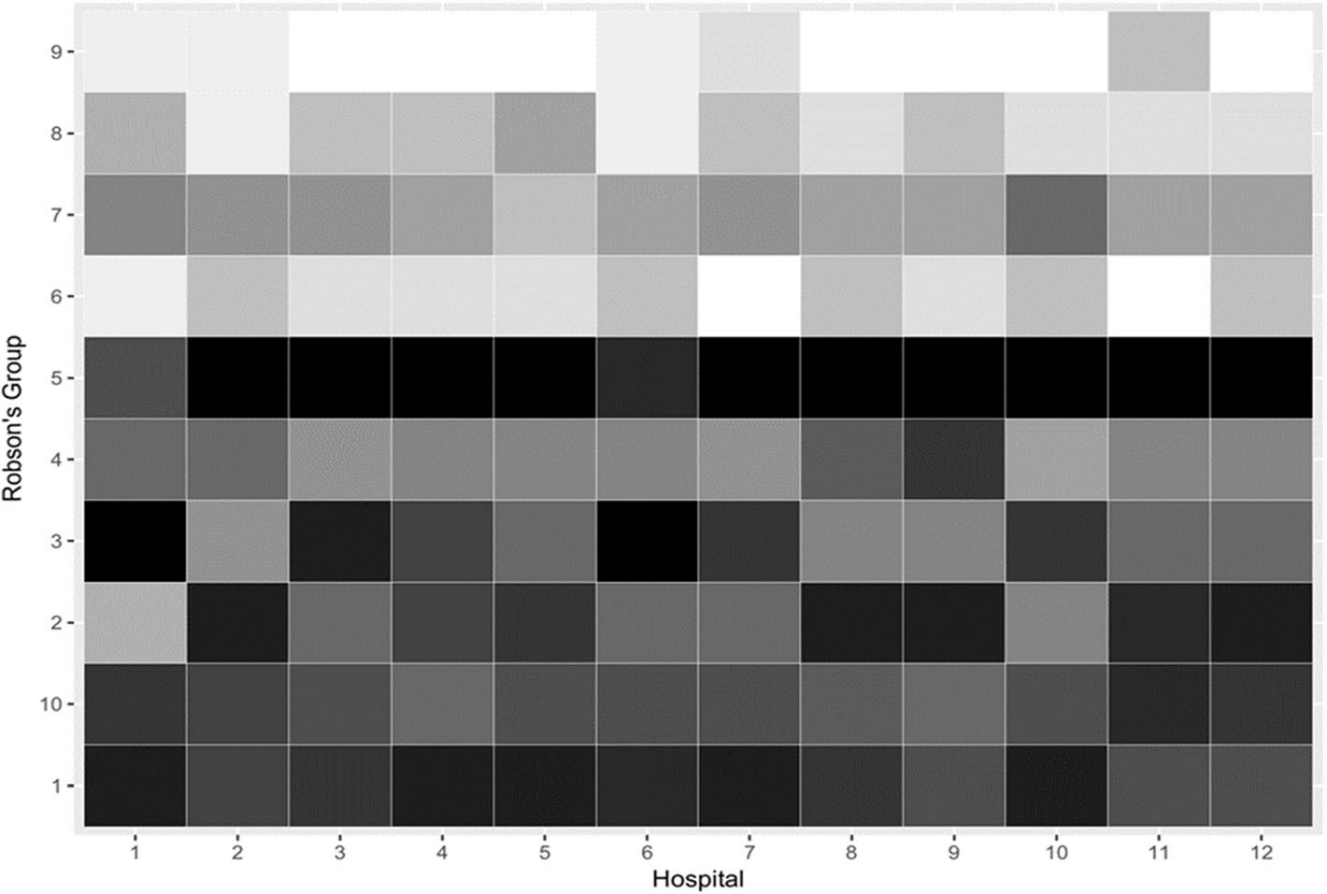
Heat map showing Robson’s 10 group classification by participating hospitals

**Fig. 4:**
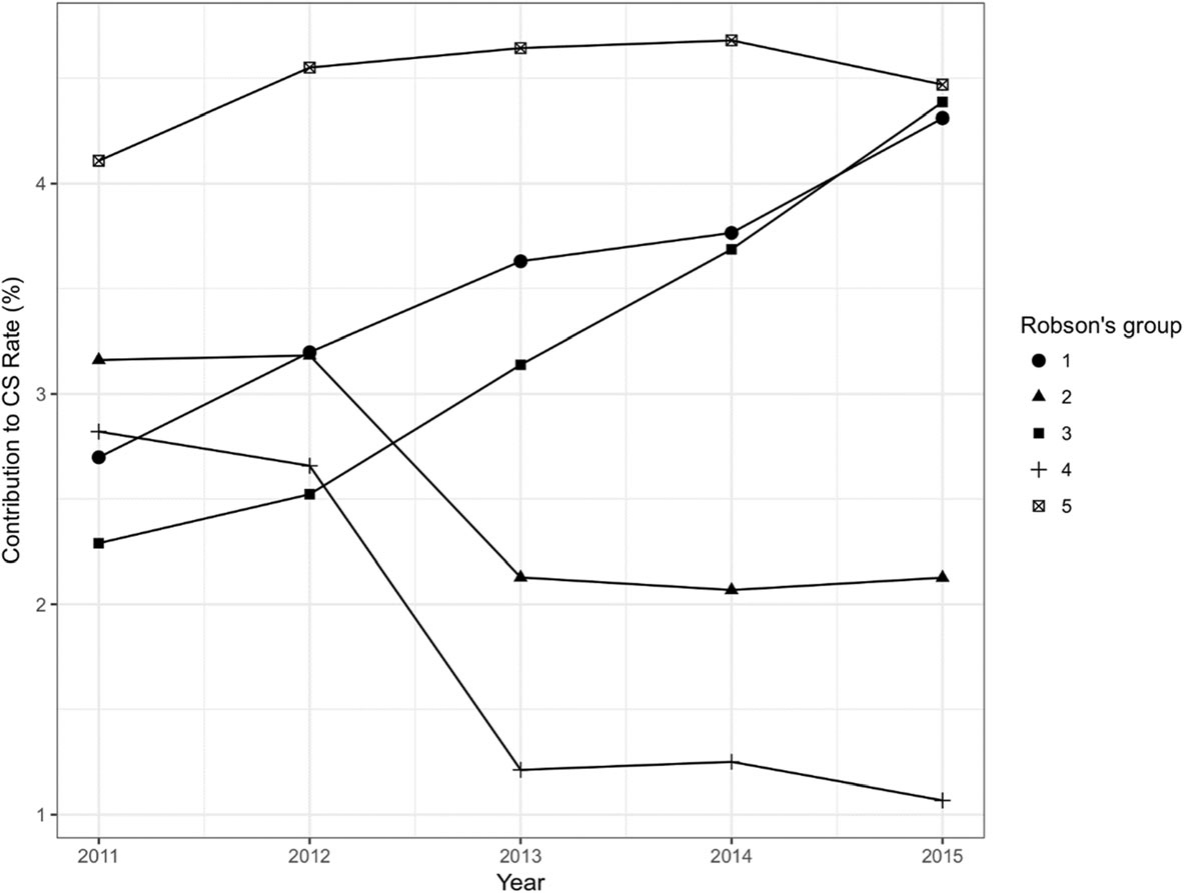
Trends of CS rate by Robson’s 10 classification

The rank order of Robson group contribution to the overall CS rate within each of the 12 hospitals is shown as a heat map in Fig. 3. The rank order is shaded from the highest rank (black) to the lowest rank (white). There are some visually obvious commonalities across the hospitals. Robson group 5 (Multiparous, singleton, cephalic, ≥ 37 weeks, with a previous CS) which made the greatest contribution to the aggregate CS rate, made the greatest contribution to the CS rate in 10 of the 12 hospitals. The two exceptions were hospitals one and six, which in both cases had Robson group 3 (Multiparous women, singleton, cephalic, ≥ 37 weeks, without a previous CS, spontaneous labour) ranked highest. These results may reflect data quality issues which are discussed later. The heatmap’s shading of Robson groups 1 and 10 show generally homogenous ranking with respect to their contributions to the CS rate. Robson group 1 (Nulliparous, singleton, cephalic, ≥ 37 weeks, spontaneous labour) for instance was generally ranked between 2nd and 4th place; and Robson group 10 (Singleton, cephalic, < 37 weeks) was generally ranked between the 4th and 5th place. The within hospital ranking generally accords with the aggregate hospital ranking, although in the aggregated hospital rankings, Robson group 3 (Multiparous women, singleton, cephalic, ≥ 37 weeks, without a previous CS, spontaneous labour) is ranked 3rd, whereas the heatmap indicates much greater between hospital heterogeneity. The relatively smaller contribution of Robson groups 6, 7, 8 and 9 is clear in the heatmap and accords with the aggregated hospital data in Table 2 (column E). Looking at the data over time, it becomes possible to look at whether the contribution of each Robson group to the overall CS rate is stable or changing. Figure 4 shows Robson groups one to five, which represent five of the top six contributors to the CS rate. A table of all the Robson’s group over time is provided as supplemental data in Table 3. Robson group 5 (Multiparous, singleton, cephalic, ≥ 37 weeks, with a previous CS) makes the greatest contribution to the overall CS rate in every year. However, Robson group 1 (Nulliparous, singleton, cephalic, ≥ 37 weeks, spontaneous labour) and group 3 (Multiparous women, singleton, cephalic, ≥ 37 weeks, without a previous CS, spontaneous labour) rise sharply from 2011 in terms of their contributions to the CS rate and effectively converge with Robson group 5 in 2015. Robson group 2 (Nulliparous, singleton, cephalic, ≥ 37 weeks, induced labour or CS before labour) and group 4 (Multiparous, singleton, cephalic, ≥ 37 weeks, without a previous uterine scar, induced labour or by CS before labour) show a decline in contribution in 2013 and then plateau.

**Table 3:** Caesarean sections / Total deliveries and Robson’s group contribution to the overall CS rate for eleven State and one Federal Territory hospitals in Malaysia in each year, 2011–2015

Robson’s Groups	2011		2012		2013		2014		2015	
	CS / Total deliveries	Contribution to overall C/S rate	CS / Total deliveries	Contribution to overall C/S rate	CS / Total deliveries	Contribution to overall C/S rate	CS / Total deliveries	Contribution to overall C/S rate	CS / Total deliveries	Contribution to overall C/S rate
Group 1	3431/23582	2.7	3725/23516	3.2	4148/20400	3.8	4917/24173	3.9	5890/27241	4.5
Group 2	4021/12882	3.2	3707/10243	3.2	2432/10958	2.2	2701/11402	2.2	2906/10115	2.2
Group 3	2911/44148	2.3	2937/41900	2.5	3587/36242	3.3	4815/43747	3.8	5993/48851	4.6
Group 4	3587/18418	2.8	3095/13147	2.7	1384/14934	1.3	1632/15783	1.3	1459/12447	1.1
Group 5	5225/9817	4.1	5299/9788	4.6	5306/9598	4.9	6112/10727	4.9	6106/11166	4.7
Group 6	1289/1411	1.0	1193/1292	1.0	1037/1118	0.9	1279/1400	1.0	1322/1436	1.0
Group 7	1883/2165	1.5	1728/1967	1.5	1648/1874	1.5	1841/2114	1.5	1991/2241	1.5
Group 8	1224/2422	1.0	1218/2345	1.0	1217/2370	1.1	1372/2664	1.1	1498/2726	1.1
Group 9	1053/1053	0.8	973/973	0.8	986/986	0.9	1113/1113	0.9	1187/1187	0.9
Group 10	3046/11262	2.4	3029/11276	2.6	3138/10738	2.9	3600/12061	2.9	4066/1338	3.1
Total	27,670/127160	21.8	26,904/116447	23.1	24,883/109218	22.8	29,382/125184	23.5	32,418/130738	24.8

## Discussion

CS is a lifesaving procedure and should be done for maternal and fetal indications. The Robson’s classification introduced in 2001 classifies women based on Obstetric characteristics rather than clinical indications. This study aimed to see which groups of patients in Malaysian tertiary hospitals contributed to the highest CS rates and to look at inter-hospital variations in CS rates using the Robson’s ten group classification which is not affected by differences in clinical practice. By using this classification, we were able to compare hospitals using a common standard set of variables. This study shows that the group with the highest CS rates was Group 5 (multiparous, singleton, cephalic≥37 weeks with a previous CS) and these rates remained high during the 5-year study period. Group 1, which is nulliparous term pregnancy with spontaneous labour, and Group 3, which is multiparous term pregnancy with spontaneous labour, are seen to have an increasing trend from 2011 to 2015. These two groups are relatively low risk and yet there was a steady rise in their CS rates – almost reaching the Group 5 rates in 2015. There is a well known relationship between induced labour and CS [15], but, interestingly we also noticed a decrease in CS trend in Group 2 (Nulliparous induced term pregnancy or elective CS) and Group 4 (Multiparous induced term pregnancy or elective CS). In 2000, the Term Breech Trial Collaboration [16] suggested planned CS is better than planned vaginal births for term breech babies which would give rise to an increase in CS rates. In our data, the overall contribution to CS rates by Group 6 (nulliparous breech) and Group 7 (multiparous breech) was only 1 and 1.5%, respectively, and this trend remained the same over the 5-year study period. Across the 12 hospitals, the CS rates ranged from 18.8 to 31.5%. In 10 of the 12 hospitals, Group 5 contributed the most to overall CS rates. Variation was seen in hospitals 1 and 6 where Group 3 contributed to an overall higher CS rate. Generally, the groups that contributed the least to the overall CS rates across the 12 hospitals were from Groups 6, 7, 8 and 9. CS is not a procedure without the risk of haemorrhage, infection and thrombosis. Assessment for induction of labour should follow guidelines and women with previous CS should have a trial of labour. Caesarean section audits should become the norm.

## Conclusions

The rise in CS reflects a globally disturbing trend, and changes in policy and training that creates a uniform standard across hospitals should be considered. Direct specialist involvement in the decisions regarding delivery in both the antepartum and intrapartum periods is important to reduce the CS rates. Their involvement is important because the reproductive future of a woman is determined by the mode of delivery of her first pregnancy. The patient should be involved in decision making after being fully informed of the facts and risks. Robson classification has made it possible to gauge rising CS rates accurately. We recommend it to be adopted as an evidenced- based tool to assess the CS rates and support remedial action to reduce the CS rates in groups 1, 3 and 5 in Malaysia. Processes to ensure good data capture have a significant role to play in ensuring the quality of the research. However, particularly in a middle-income country setting, routine data capture is particularly challenging, because it is seen by front-line staff as a diversion of limited resources away from already stretched clinical services. Overcoming this issue without depleting resources in clinical services is worthy of further investigation.

### Limitations

The Malaysian National Obstetrics Registry collects data from 14 tertiary hospitals. Only 12 hospitals were included in this study because there were some concerns about the completeness of elements of the data from the two excluded hospitals. While missing data in the order of 2.6% of deliveries is non-trivial, it remains a relatively small quantity in a database of 625,000 deliveries. Routinely collected administrative data tends to have higher rates of missing values when compared with data collected in specifically approved projects of short duration, because additional resources are often made available to short duration projects to improve the completeness of the data collection. A comparison of results in the Malaysian NOR data with the example of reporting Robson’s classification studies from the WHO suggests the need for further research to investigate potential anomalies [17].

## Data Availability

The legal data management requirements of the Ministry of Health, Malaysia do not allow data to be placed on an open access facility but we encourage collaborative research. The data is available on request for legitimate research. The data release form is available on the Malaysian NOR website: http://www.acrm.org.my/nor/

## Abbreviations

CS: Caesarean section
NOR: National Obstetrics Registry
WHO: World Health Organization
